# Assessment of Dose-Dependent Effects of 1064 nm Photobiomodulation Therapy on Tenocyte- and Bursa-Derived Cell Proliferation In Vitro

**DOI:** 10.3390/jcm15072716

**Published:** 2026-04-03

**Authors:** Zachary W. Sigman, Stefan Minyayluk, Andrew K. Chow, Sophia Blaine, Mary Beth McCarthy, Mark Cote, Marco T. Di Stefano, Monica Monici, Scott A. Sigman, Augustus D. Mazzocca

**Affiliations:** 1 Division of Sports Medicine, Department of Orthopaedic Surgery, Massachusetts General Hospital, Harvard School of Medicine, Massachusetts General Brigham, Boston, MA 02114, USA; sminyayluk@mgh.harvard.edu (S.M.); akchow@mgh.harvard.edu (A.K.C.); sblaine@mgh.harvard.edu (S.B.); mbmccarthy@mgh.harvard.edu (M.B.M.); mcote2@mgh.harvard.edu (M.C.); mtdistefano@mgh.harvard.edu (M.T.D.S.);; 2 ASA Campus Joint Laboratory, ASA Research Division, Department of Experimental and Clinical Biomedical Sciences “Mario Serio”, University of Florence, 50139 Florence, Italy; 3 Orthopaedic Surgical Associates, North Chelmsford, MA 01863, USA

**Keywords:** photobiomodulation therapy, bursa-derived cells, tenocytes, proliferation, rotator cuff, orthopedics

## Abstract

**Background/Objectives**: Photobiomodulation Therapy (PBMT) is widely used in musculoskeletal rehabilitation. Although its clinical use continues to expand, the dose-dependent metabolic responses on specific musculoskeletal cell populations remain undefined. This study assessed the effects of 1064 nm PBMT on primary human tenocytes and bursa-derived cells across varying fluence and irradiance. **Methods**: Primary tenocytes and bursa-derived cells were cultured in 24-well plates and exposed to Hiro TT 1064 nm laser at fluences ranging from 1.5 to 6.0 J/cm^2^ and irradiance levels of 90 or 125 mW/cm^2^. Treatments were administered once daily for three consecutive days. Cellular activity was assessed using an XTT assay and bright field microscopy was performed to assess cell morphology and confluency. Statistical analysis was compared to evaluate dose-dependent effects. **Results**: PBMT demonstrated tissue-dependent effects on cellular metabolic activity and proliferation. In tenocytes, moderate fluence (4.5–6.0 J/cm^2^) significantly increased metabolic activity compared with control. In contrast, bursa-derived cells exhibited smaller magnitude changes, with most treatment groups demonstrating neutral or modest deviations from control. **Conclusions**: PBMT of 1064 nm wavelenght produced distinct dose-dependent responses in musculoskeletal cell types, with tenocytes demonstrating a threshold-dependent response and bursa-derived cells showing attenuated effects. These findings support the need for tissue-specific parameters when applying PBMT in clinical tendon-related applications.

## 1. Introduction

Rotator cuff tears and other tendon injuries are among the most common causes of pain and disability in orthopedics, affecting 23% of the general population [1]. Despite this high prevalence, surgical repair remains challenging due to poor tendon-to-bone integration and high retear rates [2]. Current biomedical advances are aimed towards identifying biologic therapeutics that improve rotator cuff healing, including orthobiologics such as platelet rich-plasma, bone marrow aspirate concentrate, adipose tissue, and adjunctive biophysical therapies including photobiomodulation therapy (PBMT) [3,4].

PBMT is a noninvasive treatment that aids in tissue repair by delivering near-infrared light to trigger a series of biochemical reactions at the cellular level. Specifically, when PBMT is applied to the target site, it activates the mitochondrial respiratory chain, where the enzyme cytochrome c-oxidase (CCO) absorbs the photons, generating electrons that are passed down a proton gradient, which provides the activation energy for ATP synthase [5,6]. This process enhances mitochondrial function by increasing ATP production and reducing oxidative stress, promoting cell proliferation in many musculoskeletal tissues [7]. Additionally, the current literature suggests PBMT reduces inflammation, activates satellite cells, and promotes angiogenesis to support healing [7,8]. Clinically, PBMT is used for treatment of tendon, ligament, and bursal injuries, particularly in relation to shoulder pathology, including rotator cuff repairs. Therapeutic protocols commonly use near-infrared wavelengths (810–1064 nm) with energy densities typically between 4 and 10 J/cm^2^ at the target tissue over multiple treatment sessions, for rotator cuff tears and tendinosis [8,9]. In developing our study, we chose fluence parameters ranging from 1.5 to 6.0 J/cm^2^ to largely replicate the energy settings employed in patient care, while carefully evaluating dose-dependent responses in vitro under controlled conditions. Despite these reports, skepticism persists due to inconsistent parameters including wavelength, fluence, and irradiance, with questions about the real-world efficacy of PBMT. As highlighted by Zien et al., there is still no agreement on the parameters and protocols for its clinical applications, which limit its reproducibility across musculoskeletal treatment, and is partly due to inadequate reporting or the materials and methods, which are essential for replicating individual studies and treatment [9,10]. While in vitro studies have explored PBMT effects in skeletal muscle tissue, optimal fluence and irradiation levels for the bursa and tenocyte cells remain unexplored [8].

There is a lack of research on the effects of PBMT on cell types that are important for tendon healing, such as human tenocytes and bursa-derived cells. In the present study, a 1064 nm wavelength was selected due to its enhanced tissue penetration and potential to stimulate cellular activity in musculoskeletal tissue compared to shorter near-infrared wavelengths. Current literature shows employing the 1064 nm wavelength shows efficiency in the treatment of musculoskeletal conditions including shoulder and knee disorders [11,12]. Because tendon and subacromial bursa tissues lie beneath multiple tissue layers, longer near-infrared wavelengths such as 1064 nm are particularly suited for targeting these structures due to superior penetration depth. However, despite the increasing clinical adaptation, the cellular dose response at this wavelength remains poorly defined [13,14]. To fill this gap, the goal of this preliminary study was to investigate the optimal fluence range for enhancing cellular proliferation of human tenocytes and bursa-derived cells with PBMT. We hypothesized that the varying fluences and irradiation parameters within the range of 1.5–6.0 J/cm^2^ would produce no significant difference in metabolic activity for tendon and bursal tissue compared to untreated controls.

## 2. Materials and Methods

### 2.1. Cell Culture Setup

Bursa and tendon tissue were obtained from the same patient (80 y/o, F) during a rotator cuff repair under sterile conditions, in accordance with discard IRB approval (MGB IRB#2022P000898). Tissue samples were collected by a single orthopedic surgeon using a pituitary rongeur and placed immediately into Dulbecco’s Modified Eagle Medium (DMEM; Gibco, CA, USA) within 2 h. Tissue was minced with sterile scissors and plated in 100 mm tissue culture dishes (Thermo Fisher Scientific, Waltham, MA, USA) in Dulbecco’s Modified Eagle Medium (DMEM; Gibco, CA, USA) supplemented with 10% fetal bovine serum (FBS; Gibco, CA, USA) and 1% penicillin–streptomycin. Cultures were maintained at 37 °C in a humidified incubator with 5% CO_2_ until cellular outgrowth was observed [15]. Media were replaced twice weekly until ~80% confluence, at which point cells were passaged once. Tenocytes reached ~80% confluence in approximately 5 days and bursa-derived cells in approximately 6 days. After reaching confluence again, cells were cryopreserved at 1 × 10^6^ cells per vial in cryoprotective medium (Corning, NY, USA) (90% FBS + 10% dimethyl sulfoxide [DMSO]) using a controlled-rate cooler (−1 °C/min) and stored in liquid nitrogen for 2 months.

For experiments, vials were rapidly thawed at 37 °C, diluted into complete medium, and plated in 100 mm dishes [16]. Cells were grown to ~80% confluence, then passaged. Once confluent again, cells were trypsinized and seeded into 24-well plates (Thermo Fisher Scientific, Waltham, MA, USA) at 20,000 cells per well (passage 2 cells were used for all experiments). After 24 h of recovery, experimental PBMT treatments were initiated.

### 2.2. Hiro TT Laser Treatment

All PBMT treatments were performed using a Hiro TT laser (ASA, Italy), a high-power infrared laser device equipped with a single-source 1064 nm diode emitter. The device operates in a super-pulsed emission mode, allowing for intense delivery of high peak power, while being able to maintain low average thermal load on biological tissues and cell cultures. The system delivers an average power of 10.5 W and a peak optical power of 3000 W, with each pulse composed of a pulse duration (≤100 μs), and maximum energy of 350 mJ per pulse, allowing variation in the amount of power delivered to cells.

### 2.3. Beam Characteristics

- Wavelength: 1064 nm (near-infared)
- Spot size: 10 mm diameter (0.757 cm^2^)
- Beam profile: Gaussian
- Mode of operation: Super-pulsed
- Pulse duration: ≤100 μs
- Pulse frequency: 1–2000 Hz
- Average power: 10.5 watts
- Peak optical power: 3000 watts
- Maximum pulse energy: 350 mJ

### 2.4. Irradiation Procedure

**Figure 1:**
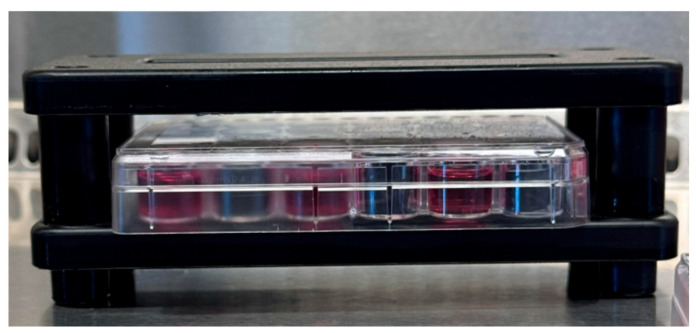
Custom support fixture used to position 24-well plates during irradiation. The fixture helps to allow consistent alignment and spacing of the plate, allowing reproducible treatment conditions across samples.

Twenty-four well plates were placed in a custom laboratory-grade plastic support, specifically designed to ensure reproducible experimental conditions. The support included a guiding groove at the top to maintain the laser handpiece (10 mm spot diameter) perpendicular to the plate surface. During irradiation, the handpiece was positioned manually while maintaining a fixed distance of 3 cm between the laser aperture and the surface of each well. This helped to ensure that the laser beam diameter matched that of a single well (Figure 1). To prevent the transmission between wells, one well was left empty between each sample, and a physical barrier was used to isolate experimental groups during treatment. This allowed for individual irradiation of each sample while minimizing cross-exposure. Such an approach is consistent with the established in vitro photobiomodulation methodologies, in which maintaining a fixed irradiance distance is critical for reproducible dosimetry, as demonstrated in previous studies [17].

### 2.5. PBMT Treatment Conditions and Group Design

PBMT application was administered at either a low (90.0 mJ/cm^2^/s) or high (125 mJ/cm^2^/s) energy density. To achieve the precise fluence delivery across all treatment groups, the required exposure time was calculated by dividing the target fluence by the dose rate to give the required time for each treatment. Cells received 1.5–6.0 J/cm^2^ per treatment corresponding to exposure durations ranging from 12 to 66 s depending on the group assignment (Table 1). These energy densities were selected based on commonly reported ranges in in vitro photobiomodulation studies, where cells are directly exposed to light without the attenuation from overlying tissues, and are known to fall within the biostimulatory window associated with the biphasic dose–response. Prior work, including Chang et al., has demonstrated that doses in the range of approximately 1–10 J/cm^2^ are effective for modulating cellular proliferation and metabolic activity in vitro [18,19].

Given the fixed laser irradiance and a well surface area of (1.9 cm^2^), this allowed for precise and reproducible energy delivery ranging from 2.85 to 11.40 J per well per treatment to cultured cells. Treatment was then administered once daily for three consecutive days. Each treatment group consisted of n = 8–10 wells per condition. The treatment samples were compared with controls maintained in the same conditions, except for the laser exposure.

### 2.6. Cell Proliferation: XTT Assay

Cellular metabolic activity was evaluated using the XTT II cell proliferation assay kit (11465015001; Roche Applied Science), following the manufacturer’s protocol. After 24 h of the final PBMT session, each well was labeled with a XTT mixture, and the plates were incubated at 37 °C for 4 h in a humidified CO_2_ incubator. Following incubation, absorbance was measured at 450 nm and a 650 nm reference with a spectrophotometer (BioTek Synergy H1 reader) and metabolic activity was analyzed. The 450 nm measurement detects the orange formazan product generated by metabolically active cells, while the 650 nm reference corrects for background optical interference from media and plate artifacts. These wavelengths are associated with the colorimetric detection of chemistry of the XTT assay and are unrelated to the 1064 nm PBMT wavelength. Additionally, because photobiomodulation directly modulates mitochondrial respiration, XTT changes likely reflect bioenergetic activation, rather than increase in cell number. Absorbance values were first blank-corrected by subtraction of background signal, then normalized to the untreated group (Group 1) to calculate relative proliferation. Each condition was tested in technical replicates (n = 8–10). 

### 2.7. Microscopy and Confluency Imaging

Brightfield microscopy was performed to assess cell confluency and morphology. Images were captured daily using a Nixon Eclipse Ts2 microscope at consistent magnification of 4× immediately before PBMT treatment and at the endpoint. These images were used to qualitatively compare changes in cell density, growth patterns, and morphology across treatment groups. While not quantitatively analyzed, these observations provided visual evidence of cellular behavior in response to varying laser fluence and irradiance. 

### 2.8. Statistical Analysis 

Raw XTT absorbance values were normalized to the untreated control group, set at 100% metabolic activity, and experimental groups were expressed as a percentage relative to this baseline. Differences between the control and treatment groups were examined with one-way ANOVA with Dunnett’s test to adjust for multiple comparisons. The effect of treatment was modeled in a two-way ANOVA with an interaction between fluence and irradiance to examine the dose–response patterns and differential change. Post hoc pairwise comparisons of treatment groups were adjusted using Tukey’s method. An alpha level of 0.05 was set for all analyses. All analyses were carried out in R (R Core Team, http://www.r-project.org, accessed on 9 February 2026).

## 3. Results

### 3.1. Dose–Response Effects of PBMT on Tenocytes

**Figure 2:**
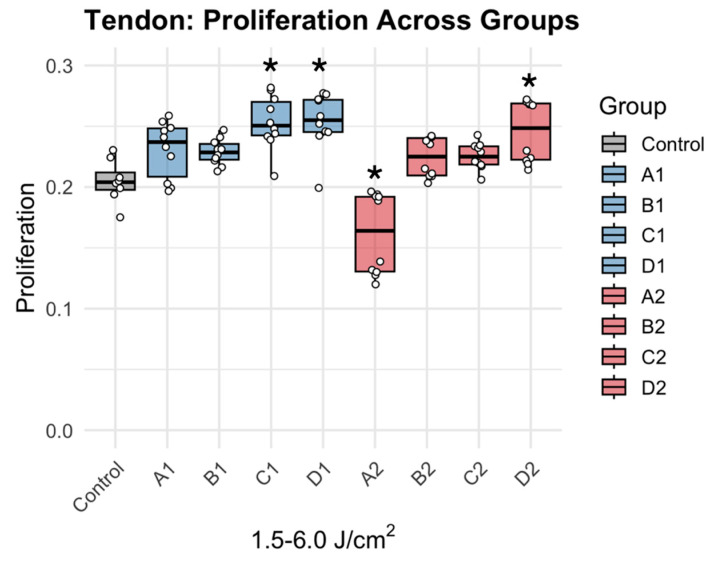
Dose dependent effects of PBMT on metabolic activity. * denotes statistically significant difference (Dunnett’s adjusted *p*-value < 0.05). Individual data points (dots) represent independent experimental replicates for each condition.

**Figure 3:**
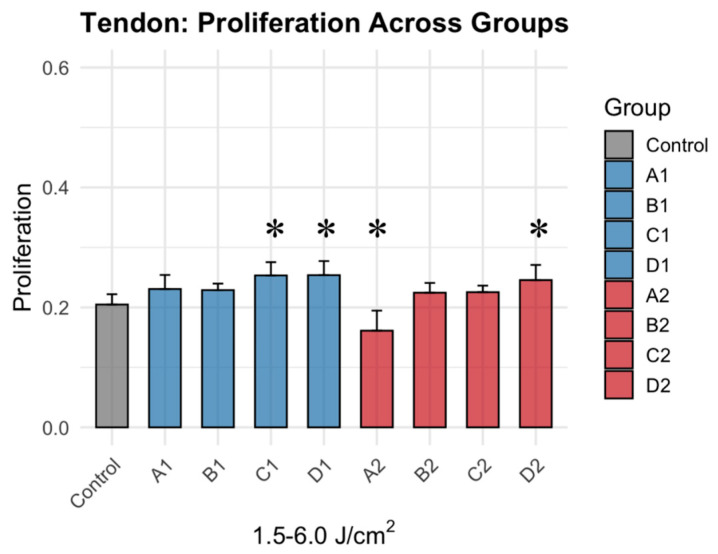
Tenocyte metabolic activity after 1064 nm PBMT across graded fluence (1.5–6.0 J/cm^2^) and irradiance conditions. * denotes statistically significance difference (Dunnett’s adjusted *p*-value < 0.05).

Photobiomodulation therapy (PBMT) produced a biphasic response in tenocyte proliferation, contingent on fluence (J/cm^2^) rather than irradiation levels (Figure 2). Moderate fluences (4.5–6.0 J/cm^2^) significantly increased metabolic activity, with groups C1 (Δ = +0.049, *p* = 0.0001), D1, (Δ = +0.0491, *p* = 0.0001), and D2 (Δ = +0.041, *p* = 0.001) showing the greatest increase in metabolic activity. Relative to control, metabolic activity increased 14–23% in groups C1 and D1 and 20% in D2 (Figure 3). Conversely, pairwise comparisons confirmed that low fluence yielded inconsistent results, with group A2 (Δ = −0.044, *p* = 0.001) demonstrating an inhibitory region, and decreased activity of 21% (Table 2). There were also several intermediate fluences (A1, B1, B2, C2) that demonstrated no significant effect during treatment, suggesting that the energy delivery was the dominant factor with tencoyte response.

### 3.2. Dose–Response Effects of PBMT on Bursa

**Figure 4:**
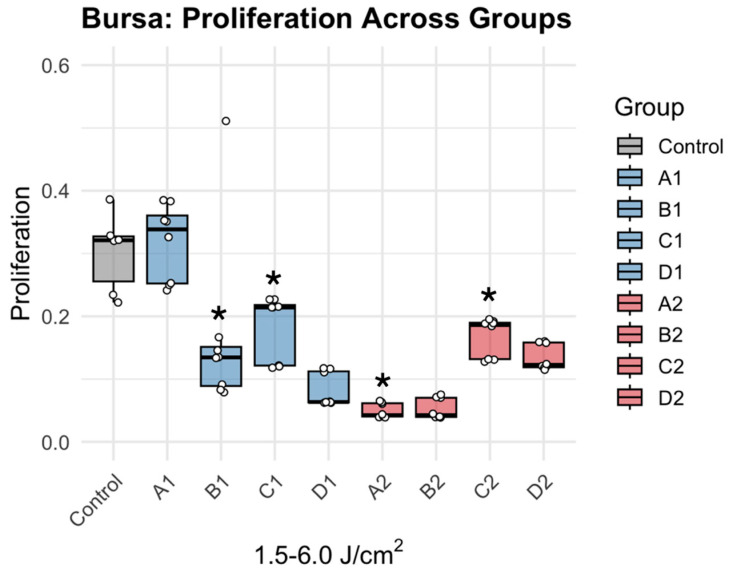
Dose dependent effects of PBMT on bursa metabolic activity. * denotes statistically significant difference (Dunnett’s adjusted *p*-value < 0.05). Individual data points (dots) represent independent experimental replicates for each condition.

**Figure 5:**
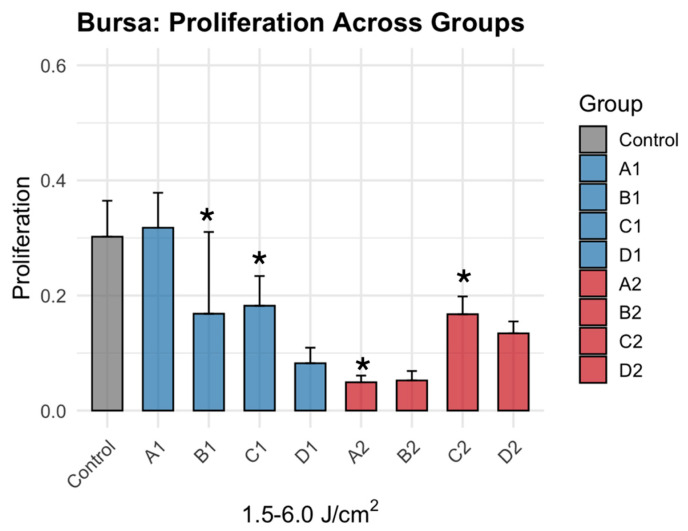
Bursa metabolic activity after 1064 nm PBMT across graded fluence (1.5–6.0 J/cm^2^) and irradiance conditions. * denotes statistically significant difference (Dunnett’s adjusted *p*-value < 0.05).

Bursa-derived cells exhibited a more attenuated and less variable response to PBMT treatment (Figure 4). Low- and high-dose treatment resulted in negligible alteration, seen by minimal changes to no changes in A1 (*p* = 0.983), and significant differences compared to control in groups B1 (Δ = 0.134, *p* = 0.001), C1 (Δ = 0.120, *p* = 0.004), A2 (Δ = 0.253, *p* < 0.0001) and C2 (Δ = 0.135, *p* = 0.0009) (Table 2). Relative to control, metabolic activity increased by 5.1% in A1 and increased approximately 12–15% across the remaining groups (Figure 5). Similar to tenocytes, no clear dependence on irradiance was observed, as responses were not consistently differentiated between low- and high-irradiance conditions at equivalent fluences.

### 3.3. Comparative Tissue Response

**Figure 6:**
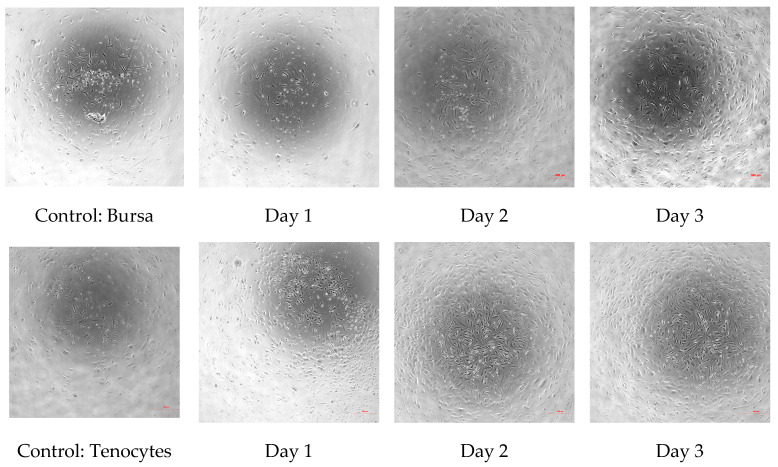
Brightfield microscopy images demonstrating temporal change in cell morphology and confluency following PBMT exposure. (Group C1).

Overall PBMT showed clear tissue-dependent effects, where tenocytes exhibited a threshold metabolic response characterized by significant stimulation at moderate fluences and inhibition at specific low-dose conditions. In contrast, bursa-derived cells demonstrated smaller magnitude but predominantly positive deviations from control across treatment conditions. Post hoc analysis using Tukey’s multiple comparisons test identified significant differences between treatment groups, with detailed pairwise comparisons provided in Table A1 (Appendix A). Across both cell types, responses appeared to be more strongly influenced by total fluence rather than irradiance, highlighting the importance of energy dose in modulating cellular activity (Figure 6).

Bursa and tenocytes were compared to baseline controls and to days 1–3 post treatment. Progressive increases in cellular confluence and density were tracked over time, with evidence of normal spindle-shaped morphology and no signs of cytotoxicity. Images were acquired at 10× magnification and are representative of (n = 8) biological replicates.

## 4. Discussion

The primary outcome of this study demonstrated the effects of PBMT at different fluences and irradiances on tenocytes and bursa-derived cells in vitro. PBMT did not exhibit biphasic, dose-dependent proliferative effects, revealing biologically opposite effects at different doses [20]. This pattern partially aligns with the Ardnt—Shulz law, which demonstrates that small doses stimulate, moderate doses inhibit, and large doses are lethal to cells; however, the response was not uniform across cell types, indicating that PBMT effects theoretically could depend not only on dose but also on the cellular phenotype and overall metabolic baseline [21,22]. The most advantageous response for tenocytes was observed at moderate fluences (3.0–4.5 J/cm^2^), aligning with the ideal doses of (1–5 J/cm^2^) mentioned in the previous literature [23]. In contrast, bursa-derived cells exhibited heightened sensitivity, showing suppression of proliferation across most conditions, particularly at higher irradiances and fluences. Bursa, particularly B1, demonstrated a comparatively greater increase in metabolic activity relative to other bursa treatment groups. This variability may reflect differences in cellular composition and baseline metabolic state, perhaps due to its heterogeneous, inflammation-susceptible cell composition being more responsive to PBMT [24].

The differential response between tenocytes and bursa-derived cells could be explained by the intrinsic cellular metabolic characteristics. Tenocytes are specialized fibroblasts which are responsible for continuous remodeling and organization of a dense, collagen-rich extracellular matrix under tensile load; the high mechanical and synthetic demands of this process require sustained ATP production. Therefore, tenocytes theoretically possess higher amounts of mitochondrial density and oxidative metabolic activity, compared to bursa-derived cells [25,26]. The extra abundance means that tenocytes potentially possess more photo-acceptors including cytochrome c oxidase, allowing for greater absorbance and, therefore, increased metabolic activity at appropriate doses. In contrast, bursa-derived cells, with potentially fewer mitochondria and photo-acceptors, are less sensitive to PBMT and could possibly experience lower or suppressed metabolic activity at higher doses [27]. The subacromial bursa’s metabolic function involves the release of bioactive molecules that are more involved with modeling immune and inflammatory response rather than absorption [28]. Therefore, the therapeutic dose of PBMT may need to be tailored to the specific density of mitochondrial photoreceptors within each cell type, as responses may appear to depend on specific density and baseline metabolic state that contribute to variability; however, these mechanisms were not directly evaluated and should be interpreted with caution. The current work was designed to identify treatment parameters that optimize cellular proliferation and metabolic activity, providing a foundation for future investigations aimed at understanding the underlying mechanistic pathways under these optimized conditions.

From a clinical standpoint, these findings may be relevant in rehabilitation protocols for rotator cuff repair. Rotator cuff surgery is complex due to poor tendon-to-bone integration, with retear rates varying from 21 to 26% depending on tear size and tissue quality [23,24]. These limitations highlight the need for adjunctive therapies, such as PBMT, aimed at improving cellular activity and enhancing the healing environment. Effective tendon-to-bone incorporation and healing necessitates active tenocyte proliferation and extracellular matrix remodeling, which facilitates collagen alignment, fibroblast proliferation, and proteoglycan synthesis [27]. PBMT’s capacity to augment tenocyte activity under moderate fluences may facilitate tendon repair by strengthening cellular activity during the remodeling stage, as demonstrated in animal models where PBMT promoted collagen organization, angiogenesis, and improved biomechanical strength [29,30]. Conversely, suppression of bursal metabolic activity and proliferation may be beneficial, as the subacromial bursa contributes to pain, fibrosis, and adhesions via inflammatory cytokines following rotator cuff repair [23]. If PBMT can selectively stimulate tendon cells while suppressing undesired bursal responses, it could clinically facilitate more effective methods to promote rotator cuff healing with rotator cuff repairs. 

In practice, PBMT is often administered peritendinously with fluences ranging from 4 to 10 J/cm^2^ for 30 s, primarily concentrating in the coracoid, upper trapezius, and anterior deltoid, utilizing a handheld wand to distribute energy within the joint [5]. This is typically conducted throughout six treatments for rotator cuff pathologies. Conversely, our in vitro model administered treatment directly to monolayer cells for three consecutive days. Although our dosing technique coincided with the lower range of clinical fluency, the absence of tissue depth and vascular alteration likely amplifies cellular sensitivity, especially seen in the bursa [31,32]. Importantly, translation of these in vitro dose–response findings to clinical practice necessitates a consideration of optical energy loss through overlying tissues. In our model, cells were directly treated at a fixed 3 cm distance without any barrier, meaning the precise fluence was applied to a monolayer of cells with no scatter and precise absorption. Generally in vivo, PBMT must travel through skin, subcutaneous tissue and the deltoid muscle before initially reaching the rotator cuff and subacromial bursa. These extra layers generally cause reflection and scattering of light which reduce the effective dose delivered to target tissues [33,34].

Additionally, all primary cells used in this study were derived from an 80-year-old-donor, likely demonstrating aged and degenerate rotator cuff pathology rather than heavy tendon physiology. The age-related alterations in mitochondrial activity, cellular proliferation capacity and inflammatory signaling could have influenced the response to photobiomodulation, and the observed dose–response relationships should therefore be interpreted within the context of geriatric tendon tissue. 

Nonetheless, the clinical application of these results requires careful attention to dosing parameters. The biphasic nature of PBMT indicates that excessive dosing could inhibit tendon healing, while insufficient dosing may not effectively promote repair. The findings from this preliminary in vitro study concentrated solely on cell proliferation with varying fluences. The relatively small sample size, particularly in the bursa-derived experiment, may have limited statistical power to detect differences between treatment conditions. Proliferation was assessed exclusively by XTT metabolic activity, which is commonly used. Other critical healing processes, including extracellular matrix deposition, collagen alignment, and mechanical strength, were not assessed. Additionally, more detailed mechanistic and metabolic analyses (mitochondrial function assays) were beyond the scope of this study due to resource constraints. Moreover, in vitro responses may not fully replicate the complex biomechanical and inflammatory environment of the shoulder, making clinical translation difficult. However, more tests and proliferation endpoints should be explored in future experiments to further clarify the therapeutic relevance of PBMT for tendon and bursal healing.

## 5. Conclusions

This study illustrates that tenocyte- and bursa-derived cells display divergent metabolic responses to PBMT; tenocytes attained maximal metabolic activity, whereas bursa-derived cells exhibited greater sensitivity, evidenced by marked suppression at higher doses. These findings underscore the necessity for tissue-specific PBMT protocols in musculoskeletal therapy. Although the underlying mechanisms remain unclear, differences in cellular phenotype and metabolic activity may contribute to the observed variability. Future research should explore the mechanisms of repair and regeneration in each cell type and expand beyond proliferation endpoints to include functional and mechanistic outcomes. Expanding this work will help refine clinical dosing protocols and clarify the therapeutic potential of PBMT in orthopedic surgery.

## Figures and Tables

**Table 1:** PBMT treatment parameters for tenocyte- and bursa-cell cultures. Experimental groups were labeled A–D to indicate increasing fluence (1.5, 3.0, 4.5 and 6.0 J/cm^2^) and the suffix ‘1’ or ‘2’ denotes the irradiance level.

Group	Fluence (J/cm^2^)	Irradiance (W/m^2^)	Irradiance Time (s)	Energy Delivered (J/well)
Control	-	-	-	-
A1	1.5	90	16.00	2.85
B1	3.0	90	33.00	5.70
C1	4.5	90	50.00	8.55
D1	6.0	90	66.00	11.40
A2	1.5	125	12.00	2.85
B2	3.0	125	24.00	5.70
C2	4.5	125	36.00	8.55
D2	6.0	125	48.00	11.40

**Table 2:** Comparisons of treatment groups to the control cell type (Dunnett’s adjustment).

	Tenocyte			Bursa	
Comparison	Difference	*p*-Value	Comparison	Difference	*p*-Value
Control-A1	0.026	0.083	Control-A1	−0.016	0.983
Control-B1	0.024	0.124	Control-B1	0.134	0.001
Control-C1	0.049	<0.001	Control-C1	0.120	0.004
Control-D1	0.049	<0.001	Control-D1	0.220	<0.001
Control-A2	−0.044	<0.001	Control-A2	0.253	<0.001
Control-B2	0.020	0.283	Control-B2	0.250	<0.001
Control-C2	0.021	0.242	Control-C2	0.135	0.001
Control-D2	0.041	0.001	Control-D2	0.168	<0.001

## Data Availability

The data presented in this study are available on request from the corresponding author. The data are not publicly available due to institutional and research-related restrictions.

## Abbreviation

The following abbreviation is used in this manuscript:

PBMT	Photobiomodulation therapy

## Appendix A

Additional pair wise comparisons of each treatment group.

**Table A1:** Pairwise comparison groups by cell type (Tukey’s adjustments).

	Tenocytes			Bursa	
Comparison	Difference	*p*-Value	Comparison	Difference	*p*-Value
A1-B1	0.002	1.000	A1-B1	0.150	0.0002
A1-C1	−0.023	0.3013	A1-C1	0.136	0.0009
A1-D1	−0.023	0.2751	A1-D1	0.236	<0.0001
A1-A2	0.069	<0.001	A1-A2	0.269	<0.0001
A1-B2	0.006	0.9985	A1-B2	0.265	<0.0001
A1-C2	0.005	0.9995	A1-C2	0.150	0.0002
A1-D2	−0.015	0.7953	A1-D2	0.184	<0.001
B1-C1	−0.025	0.2139	B1-C1	−0.014	0.9998
B1-D1	−0.025	0.1931	B1-D1	0.086	0.1027
B1-A2	0.068	<0.001	B1-A2	0.119	0.0051
B1-B2	0.004	0.9998	B1-B2	0.116	0.0071
B1-C2	0.003	1.000	B1-C2	0.001	1.0000
B1-D2	−0.017	0.6864	B1-D2	0.034	0.9478
C1-D1	−0.001	1.000	C1-D1	0.100	0.0325
C1-A2	0.092	<0.001	C1-A2	0.133	0.0012
C1-B2	0.029	0.0807	C1-B2	0.130	0.0017
C1-C2	0.028	0.1007	C1-C2	0.015	0.9997
C1-D2	0.008	0.9929	C1-D2	0.048	0.7553
D1-A2	0.093	<0.001	D1-A2	0.033	0.9545
D1-B2	0.029	0.0711	D1-B2	0.030	0.9739
D1-C2	0.028	0.0891	D1-D2	−0.085	0.1097
D1-D2	0.008	0.9897	D1-D2	−0.052	0.6714
A2-B2	−0.063	<0.001	A2-B2	−0.003	1.0000
A2-C2	−0.064	<0.001	A2-C2	−0.118	0.0056
A2-D2	−0.084	<0.001	A2-C2	−0.085	0.1097
B2-C2	−0.001	1.000	B2-C2	−0.115	0.0077
B2-D2	−0.021	0.4005	B2-D2	−0.082	0.1393
C2-D2	−0.020	0.4581	C2-D2	0.033	0.9545
